# A Novel Key Features Screening Method Based on Extreme Learning Machine for Alzheimer’s Disease Study

**DOI:** 10.3389/fnagi.2022.888575

**Published:** 2022-05-25

**Authors:** Jia Lu, Weiming Zeng, Lu Zhang, Yuhu Shi

**Affiliations:** ^1^Laboratory of Digital Image and Intelligent Computation, Shanghai Maritime University, Shanghai, China; ^2^Basic Experiment and Training Center, Shanghai Maritime University, Shanghai, China; ^3^College of Information Engineering Shanghai Maritime University, Shanghai, China

**Keywords:** fMRI, brain functional connectivity, extreme learning machine, AD, KFS-ELM

## Abstract

The Extreme Learning Machine (ELM) is a simple and efficient Single Hidden Layer Feedforward Neural Network(SLFN) algorithm. In recent years, it has been gradually used in the study of Alzheimer’s disease (AD). When using ELM to diagnose AD based on high-dimensional features, there are often some features that have no positive impact on the diagnosis, while others have a significant impact on the diagnosis. In this paper, a novel Key Features Screening Method based on Extreme Learning Machine (KFS-ELM) is proposed. It can screen for key features that are relevant to the classification (diagnosis). It can also assign weights to key features based on their importance. We designed an experiment to screen for key features of AD. A total of 920 key functional connections screened from 4005 functional connections. Their weights were also obtained. The results of the experiment showed that: (1) Using all (4,005) features to diagnose AD, the accuracy is 95.33%. Using 920 key features to diagnose AD, the accuracy is 99.20%. The 3,085 (4,005 - 920) features that were screened out had a negative effect on the diagnosis of AD. This indicates the KFS-ELM is effective in screening key features. (2) The higher the weight of the key features and the smaller their number, the greater their impact on AD diagnosis. This indicates that the KFS-ELM is rational in assigning weights to the key features for their importance. Therefore, KFS-ELM can be used as a tool for studying features and also for improving classification accuracy.

## Introduction

Alzheimer’s disease (AD) is a neurodegenerative disease. Compared to cognitively normal individuals, the AD patient’s brain undergoes morphological or functional changes. For example, Kazemifar et al. (2014) found significant atrophy of the temporal cortex in AD patients. Supekar et al. (2008) used resting-state fMRI to find reduced local efficiency of functional brain networks in AD patients. Buckner et al. (2009) used resting-state fMRI data to find that the distribution of core nodes in the functional brain network overlaps highly with the brain regions where Aß amyloid is deposited in AD patients. It means that the brain connection hub area is vulnerable to receive attacks. Zhou et al. (2012) used resting-state functional brain networks to predict the course of developmental changes in AD pathology. These studies above not only provide experimental evidence for the brain region disconnection hypothesis of AD from the perspective of functional integration, but also provide an explanation for the abnormalities in functional integration of the AD brain.

The AD Neuroimaging Initiative (ADNI), led by Dr. Weiner, was launched in 2004. This is a long-term research plan jointly composed of several institutions. It integrates the database of multi-center and cross-disciplinary longitudinal studies carried out by various methods. Such as clinical cognitive function evaluation, neuroimaging examination, and detection of molecular biological markers of cerebrospinal fluid and blood (Susanne et al., 2005). Its primary goal is to explore the patterns of relationships between clinical, cognitive, imaging, genetic and biomarker features as the disease progresses. It also has the goal of establishing standardized methods for imaging/biomarker collection and analysis, and ultimately for use in clinical research.

In 2004, Huang et al. (2004) proposed the Extreme Learning Machine (ELM). ELM generates random input weights and uses the Moore-Penrose pseudo-inverse (MPP) to calculate the output weights. It is a feed-forward neural network based on randomization(RFNN). The RFNN was introduced by Schmidt et al. (1992) and Suganthan and Katuwal (2021). Methods using random input weights and MPP also include random vector function linked neural networks (RVFL) (Pao et al., 1994), and a method proposed by Guo et al. (1995).

The ELM be widely used in many fields such as disease diagnosis, traffic sign recognition, image quality assessment and so on (Chyzhyk et al., 2015; Huang et al., 2016; Wang et al., 2016). ELM strives to solve the research problems in machine learning fields such as regression, classification, supervised learning and unsupervised learning under a single framework (Huang et al., 2014). From the perspective of learning efficiency, ELM is concise to implement, with extremely high learning speeds and less human intervention. From the perspective of theoretical studies, ELM can still maintain SLFN’s interpolation ability (Huang et al., 2016), general approximation ability (Huang and Chen, 2007) and classification ability (Huang et al., 2012) even in the case of randomly generating hidden layer neuron parameters. From the perspective of structural risk minimization, the VC dimensionality (Vapnik-Chervonenkis dimensionality) of ELM depends on the number of neurons in the hidden layer (Liu et al., 2012). The size of the VC dimensionality can be controlled by adjusting the number of neurons in the hidden layer of ELM, to make a compromise between training error and model complexity, and get the optimal generalization performance. ELM has also been extended to a deep learning model (Tang et al., 2015; Kim et al., 2017), and made a lot of research results.

In recent years, RFNN has also been gradually used for AD studies based on medical images. Sharma et al. (2021) proposed that the FAF-DRVFL method achieved 86.67% accuracy in classifying AD with CN. Malik et al. (2022) proposed the IFRVFL method for diagnosing AD. Its performance is better than standard ELM and RVFL. Lama and Kwon (2021) proposed the ElM+Graph embedding method achieved 90.93% accuracy in classifying AD, MCI, CN. Nguyen et al. (2019) proposed the MVPA+ELM method achieved 98.86% accuracy in classifying AD, MCI, CN. The RFNN is often used as a classifier in these studies. It is mainly used to process selected low-dimensional features. Few studies have used RFNN as a feature screening method.

The ELM classifier has redundant hidden layer nodes when the number of hidden layer nodes is large enough. Rong et al. (2008) proposed the method Pruned-Extreme Learning Machine (P-ELM), as well as Miche et al. (2010, 2011) improved the P-ELM method, which was used to pruning of the hidden layer nodes with the aim of making the ELM classifier more compact, with high speed and more robustness. But it cannot improve the accuracy of the classifier. In ELM classifiers with high feature dimensionality, some input nodes have no positive effect on classification. Useless input nodes are removed using the idea of P-ELM. It can screen features that have an impact on the classification. It may even improve the accuracy of the classifier.

We analyzed the state of machine learning techniques in diagnosing AD (Tanveer et al., 2020). We found no method to prune the network to extract key features. Some of the work involving AD feature extraction (Bi et al., 2018; Richhariya et al., 2020; Hao et al., 2021; Sadiq et al., 2021) have achieved good results in terms of classification accuracy, but none have assigned weights to the importance of these features. In this study, we propose a novel key features screening method based on extreme learning machine (KFS-ELM) to screen key features of AD. It is a data-driven approach that is not based on empirical assumptions or prerequisites. It prunes the ELM classifier to determine the relationship between each feature and AD, screens the key features, and identifies their importance. This will make the key features more intuitive, facilitate the study of the patterns behind AD, and benefit the construction of better classifiers to diagnose AD. The KFS-ELM can be used not only for studying AD, but also for other research fields where the studied subjects have a high dimension of features.

The rest of this article is organized as follows. The section “Materials and Methods” describes the “Brain Functional Connectivity Network,” “ELM,” and “KFS-ELM method.” The section “Experiment” introduces the experimental procedure, experimental environment, data preparation, screening of key features by the KFS-ELM method, and validation of key features. The section “Results” presents the streamlining ability of the KFS-ELM method, the distribution of the screened key features, and the effect of the key features on the classification of the ELM classifier. The section “Discussion” discusses the limitations of the ELM method and the advantages of the KFS-ELM method, and analyzes the key features. The section “Conclusion” summarizes the work of this study.

## Materials and Methods

### Brain Functional Connectivity

Brain functional connectivity network is a mathematical representation defined by a set of nodes and edges (Rubinov and Sporns, 2010; Liu et al., 2016). These nodes represent brain regions on different scales. The temporal correlations (functional connectivity) between the fMRI time course of these nodes form the edge of the brain’s functional network. The smaller the size of a node, the greater the number of nodes and edges. The more complex the described pattern of neural activity in the brain, the more difficult it is to calculate and analyze. Researchers often divide the brain into regions or nodes by using atlas. automated anatomical labeling (AAL) (Tzourio-Mazoyer et al., 2002) is one of the most commonly used atlas. Templates is one of the most commonly used templates. AAL divides the brain into 116 regions, including 90 regions of the cerebrum and 26 regions of the cerebellum. The feature measures adopted in this paper is the whole cerebrum functional connectivity network. It includes 4005 functional connectivity features.

### Extreme Learning Machine

Extreme learning machines belong to single hidden layer feed forward neural networks (SLFNs) and have the characteristics of single hidden layer neural networks. (1) Implement complex nonlinear mapping directly from the input layer. (2) It can provide appropriate classification model for large category data sets. Compared with other single hidden layer neural network models, the speed of model training and classification is faster. Huang and Babri (1998) indicated that the input layer weights and hidden layer bias values of other SLFNS networks need to be iteratively adjusted to fit the current training data. When there are a large number of hidden layer nodes, this calculation can lead to significant computational time consumption (Huang and Chen, 2008; Huang et al., 2011). At the same time, since gradient descent has become an effective method to solve SLFNs, this method not only limits the solving speed, but also can easily fall into the local minimum from the calculation principle of the calculation method. Aiming at the above problems, Huang et al. (2006) proposed the algorithm of extreme learning machine. It transformed the iterative solution method into the solution method of linear equations by randomly specifying the weight and bias values of the input layer, and finally obtained the analytical solution of the network. It can be quickly solved on the premise of ensuring the accuracy of calculation.

Extreme learning machine can be described as: given N arbitrary samples {*X*_*i*_,*t*_*i*_}, *X*_*i*_=${\left[x_{i1},\;x_{i2},\;\cdots ,\;x_{in}\right]}^{T}$ ∈ *R^n^*,*t*_*i*_=${\left[t_{i1},\;t_{i2},\;\cdots ,\;t_{im}\right]}^{T}$ ∈ *R^m^*. For a single hidden layer neural network with L hidden layer nodes, it can be expressed as


(1) $$\sum_{i=1}^{L}\beta_{i}\,g\,\left(W_{i}\cdot X_{j}+b_{i}\right)=O_{j},i=1,\cdots ,L\,j=1,\ldots ,N$$

Where *g*(*x*) is the activation function. *W*_*i*_=[*w*_*i*,1_,*w*_*i*,2_,…,*w*_*i*,*n*_] is input weight. β_*i*_=${\left[\beta_{i,1},\;\cdots ,\;\beta_{i,m}\right]}^{T}$ is output weight, *b_i_* is the bias of the *ith* hidden layer node. *O*_*j*_=${\left[o_{j,1},\;\cdots ,\;o_{j,m}\right]}^{T}$ is output of the sample *X_j_*. The goal of single-hidden layer neural network learning is to minimize the output error, can be represented as


(2) $$\sum_{i=1}^{N}||O_{i}-T_{i}||=0,i=1,\ldots ,N$$

There exist β_*i*_, *W_i_* and *b_i_*, such that


(3) $$\sum_{i=1}^{L}\beta_{i}\,g\,\left(W_{i}\cdot X_{j}+b_{i}\right)=T_{j},j=1,\ldots ,N$$

It can be expressed in matrix form


(4) H⁢β=T

Where *H* is the output of the hidden layer node, β is the output weight, and *T* is the expected output.


(5) $$H=\left(W_{1},\ldots ,W_{L},b_{1},\ldots ,b_{L},X_{1},\ldots ,X_{L}\right)$$

$$={\left[\begin{array}{ccc} g\,\left(W_{1}\cdot X_{1}+b_{1}\right) & \cdots & g\,\left(W_{L}\cdot X_{1}+b_{L}\right)\\ \vdots & \cdots & \vdots \\ g\,\left(W_{1}\cdot X_{N}+b_{1}\right) & & g\,\left(W_{L}\cdot X_{N}+b_{L}\right) \end{array}\right]}_{N\times L}$$

(6) $$\beta ={\left[\begin{array}{c} \beta_{1}^{T}\\ \vdots \\ \beta_{L}^{T} \end{array}\right]}_{L\times m},T={\left[\begin{array}{c} T_{1}^{T}\\ \vdots \\ T_{N}^{T} \end{array}\right]}_{N\times m}$$

In the ELM algorithm, *W_i_* and *b_i_* are randomly determined, and the output matrix *H* of the hidden layer is uniquely determined. The training of the single-hidden layer neural network can be transformed into adding a linear system, Equation (4). And the output weight β can be determined by Equation 7.


(7) $$\hat{\beta }=H^{†}\,T$$

Where *H*^†^ is the Moore-Penrose inverse of *H*. and the solution norm of $\hat{\beta }$ is minimal and unique. We solve for $\hat{\beta }$ to construct ELM.

In this paper, the ELM classifier is constructed by dividing the data set into three sets: training set, validation set, and test set. The training set is used to build enough classifiers. The validation set is used to verify the accuracy of each classification, and find the best ELM classifier. As the input weights of the ELM classifier are randomly generated, its classification accuracy will also vary randomly. To ensure that the classifier has high accuracy, a sufficient number of ELM classifiers need to be trained, until the average accuracy of all classifiers converges. The number of classifiers constructed is *Loop*, and the average accuracy of ELM classifiers is $\bar{A\,c\,c\,u\,r\,a\,c\,y}$. The value of the variable *Loop* should satisfy the constraint of Equation 9, where the parameter *p* is the allowed fluctuation of the average accuracy. The loop calculation produces 2*Loop accuracies, and the absolute value of the difference between the average accuracies of any consecutive *Loop* times should be less than or equal to parameter *p*. The 2*Loop ELM classifiers are validated with the validation set, and the classifier with the highest accuracy is the optimal classifier we are looking for. In this experiment *p* = 0.15%.


(8) $$\bar{A\,c\,c\,u\,r\,a\,c\,y}=\frac{\sum_{i=1}^{L\,o\,o\,p}A\,c\,c\,u\,r\,a\,c\,y_{i}}{L\,o\,o\,p}$$

(9) $$\left|\frac{\sum_{i=n}^{L\,o\,o\,p+n-1}A\,c\,c\,u\,r\,a\,c\,y_{i}}{L\,o\,o\,p}-\frac{\sum_{i=1}^{L\,o\,o\,p}A\,c\,c\,u\,r\,a\,c\,y_{i}}{L\,o\,o\,p}\right|\leq p,$$

n∈(2,3,…,L⁢o⁢o⁢p+1)

### KFS-ELM Method

The main idea of the KFS-ELM algorithm lies in pruning the ELM classifier. Removing input nodes and hidden layer nodes (including the weights connected to them) which have no positive impact with the classification, and keeping the accuracy of the ELM classifier from decreasing on the training and validation sets. The features corresponding to the input layer nodes of the pruned classifier are considered as key features. They are strongly related to the classification. **Definition 1:** *W^i^* is the diagonal matrix, *i* ∈ [1,…,*n*],*n* is the dimensionality of the subject’s features. *W^i^* satisfies the constraints of *rank*(*W^i^*) = *i*, *rank*^(*^) denotes the rank of the matrix. **Definition 2:** β*^j^* is the diagonal matrix, *j* ∈ [1,⋯,*L*], *L* is the number of hidden layer nodes of the ELM classifier. β*^j^* satisfies the constraints of rank(β)j=j.

In the ELM classifier the output weight $\hat{\beta }$, the input weight *W*, and the bias *b* are known quantities. The output weights after pruned can be expressed as $\beta^{j}\cdot \hat{\beta }$. The input weights can be expressed as *W*⋅*W^i^*. The key feature matrix of the subject can be expressed as *W^i^*⋅*X*. One *W^i^* corresponds to a group of key features. The pruned ELM classifier can be described by Equation (10) as


(10) $$f\,\left(W^{i},\beta^{j}\right)=g\,\left(W\cdot W^{i}\cdot X+b\right)\cdot \beta^{j}\cdot \hat{\beta }=O$$

*g*(*) is the activation function. *X* is the input matrix consisting of the features of the input data set (training set, validation set and test set). *X* = [*X*_1_,⋯,*X*_*N*_], where *X_i_* denotes the feature vector of the *ith* subject, and there are *N* subjects. *O* is the output matrix.*O* = [*O*_1_,⋯,*O*_*N*_], *O_i_* is the label vector output by the ELM classifier, corresponding to a subject. *O*_*i*_=${\left[o_{i,1},\;\cdots ,\;o_{i,m}\right]}^{T}$, there is only one *o*_*i*,*j*_=1,*o*_*i*,*j*_ ∈ *O*_*i*_, the others *o*_*i*,*j*_ = 0. *T* = [*T*_1_,⋯,*T*_*N*_] is the set of labels corresponding to the data set, where *T_i_* denotes the label vector corresponding to the *ith* subject. *T*_*i*_=${\left[t_{i,1},\;\cdots ,\;t_{i,m}\right]}^{T}$, there is only one *t*_*i*,*j*_=1,*t*_*i*,*j*_ ∈ *T*_*i*_, the others *t*_*i*,*j*_=0. The accuracy of the ELM classifier can be expressed as Equation 11. ∥*∥ denotes the Modulus of the vector.


(11) $$h\,\left(O\right)=1-\frac{1}{N}\,\sum_{i=1}^{N}\frac{∥T_{i}-O_{i}∥}{\sqrt{2}}$$

In Equation 10, *W^i^* and β*^j^* are the variables to be solved, and the other parameters are known quantities. *W^i^* should satisfy inequality (Equation 12). β*^j^* should satisfy inequality (Equation 13).


(12) $$\left\{ \begin{array}{c} h\,\left(f\,\left(\beta^{j},W^{i}\right)\right)>h\,\left(f\,\left(\beta^{j},W^{i-1}\right)\right)\\ h\,\left(f\,\left(\beta^{j},W^{i}\right)\right)>h\,\left(f\,\left(\beta^{j},W^{i+1}\right)\right) \end{array}\right.$$

(13) $$\left\{ \begin{array}{c} h\,\left(f\,\left(\beta^{j},W^{i}\right)\right)>h\,\left(f\,\left(\beta^{j-1},W^{i}\right)\right)\\ h\,\left(f\,\left(\beta^{j},W^{i}\right)\right)>h\,\left(f\,\left(\beta^{j+1},W^{i}\right)\right) \end{array}\right.$$

Pruning an ELM classifier obtains a *W^i^*, which corresponds to a set of key features. Pruning enough ELM classifiers will obtain enough *W^i^*. The merge set of W*=∑*W^i^* correspond to the complete key features. It should be noted that the datasets used for validation W* are the training and validation sets and do not include the test set.

### KFS-ELM Algorithm Steps

1. Construct an ELM classifier using all the features from the training and validation sets.
2. Prune the input layer nodes and their corresponding output weights in the ELM classifier, which means that keeping β*^j^* constant to solve *W^i^* iteratively. The initial values of *W^i^* and β*^j^* are *nth*-order unit matrix and *Lth*-order unit matrix, respectively, where n is the feature dimensionality of the subject and m is the number of ELM hidden layer nodes. When the inequality (12) is satisfied, take *W^i^* and β*^j^* into step 3.
3. Prune the input layer nodes and their corresponding output weights in the ELM classifier, which means that keeping *W^i^* constant to solve β*^j^* iteratively. When β*^j^* satisfies inequality (13), take *W^i^* and β*^j^* into step 2. When *W^i^* and β*^j^* satisfy both inequalities (10) and inequality (11), take *W^i^* into step 4.
4. Loop through steps 1-3, find W*=∑*W^i^*. And Construct ELM classifier based on the key features corresponding to W*. When the accuracy of the ELM classifier is no longer increasing, the calculation ends. The features corresponding to the final W* are the key features screened by the KFS-ELM method.

## Experiment

### Flow of the Experiment

**FIGURE 1:**
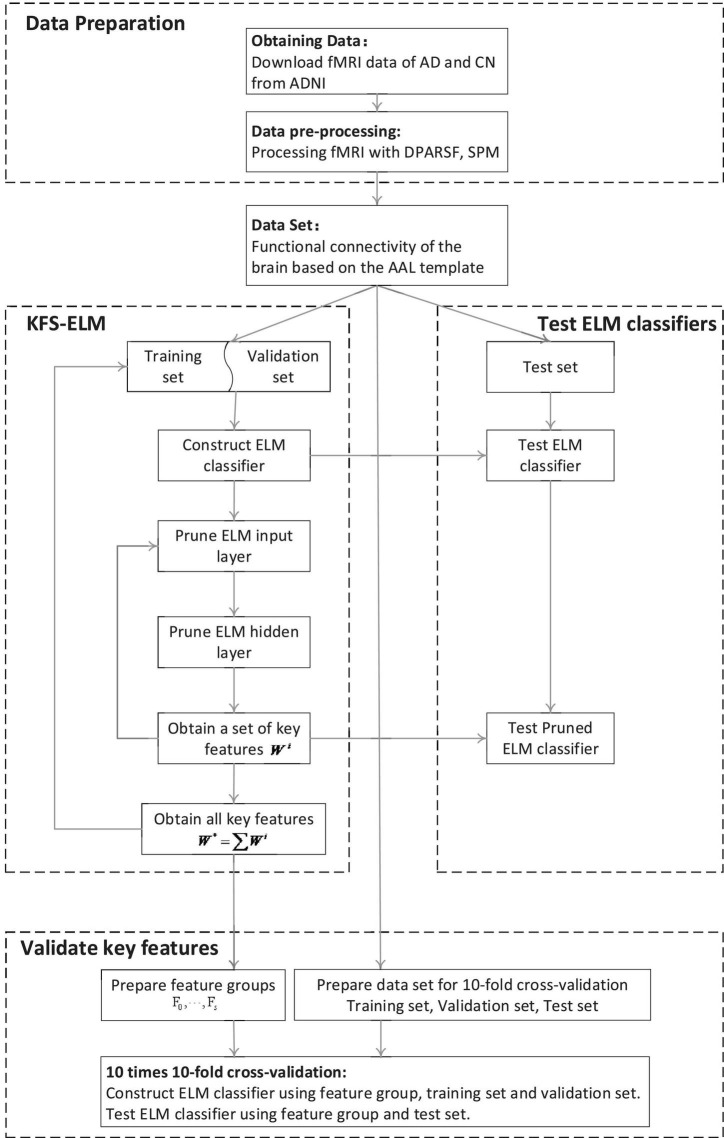
Flow of the experiment.

This experiment consists of three parts: data preparation, screen features by using KFS-ELM, test ELM classifier, and validate key features (as shown in Figure 1).

1. Data preparation: fMRI data for AD and CN are acquired from the ADNI database and pre-processed using DPARSF, SPM to obtain the functional brain connectivity matrix for all subjects.
2. Screen key features using KFS-ELM: Screening for key features which are relevant to the diagnosis of AD.
3. Test ELM classifier: Test the ELM classifier before and after pruning, and observe the change in classifier performance.
4. Validate key features: compare the ability to diagnose AD using the key feature set versus the full feature set. Compare the impact of key features with different weights on diagnosing AD.

### Environment of the Experiment

All experiments run on a PC with intel core i7-8700 @ 3.20GHz, NVIDIA GeForce RTX 2080 8GB, 16GB DDR4 3600MHz, 1TB SSD.

### Data of the Experiment

The fMRI data used in this paper are all from LONI’s ADNI database ADNI2 project. It included 100 Cognitively Normal (CN) subjects, 49 females and 51 males, with a mean age and standard deviation of 74.09 ± 5.45. And it included 100 Alzheimer’s disease (AD) subjects, 44 women and 56 men, with a mean age and standard deviation of 75.07 ± 7.59. All subjects used the same acquisition parameters. Field Strength = 3.0 tesla; Flip Angle = 80.0 degree; Manufacturer = Philips Medical Systems; Matrix X = 64.0 pixels; Matrix Y = 64.0 pixels; Mfg Model = Intera; Pixel Spacing X = 3.3125 mm; Pixel Spacing Y = 3.3125 mm; Pulse Sequence = GR; Slices = 6720.0; Slice Thickness = 3.312999963760376 mm; TE = 30.000999450683594 ms; TR = 3000.0 ms; The subject data can be downloaded at http://adni.loni.usc.edu.

### Pre-processing of Data

The data pre-processing tools chosen for this experiment are ‘‘Data Processing Assistant for Resting-State fMRI Advanced Edition’’ (DPARSF 4.4 Advanced Edition^1^)(Yan and Zang, 2010) and ‘‘ Statistical Parametric Mapping’’ (SPM12^2^). Remove the first 10 time points for each subject in order to remove the phase where the subject is familiar with the MRI scanner environment at the beginning of the data scan, and where brain activity is not smooth during the noise. Slice Timing and Head Motion correction is performed for each subject, and EPI template is used for standardization. Band-pass filtering is used to obtain signals between 0.01 and 0.1Hz. After processing, the bounding box of all subjects is [-90-126-72; 90 90 108], and the Voxel size is [3 3 3]. And then detrend the signal. The functional connectivity network of the brain is extracted based on the ALL template. Finally, we obtained 100 functional connectivity matrices for each of the two categories of subjects, AD and CN.

### Grouping of Data Sets

The 200 functional connection matrices are divided into three groups: training set, validation set, and test set. The ratio of AD subjects to CN subjects within each group is 1:1.

In the KFS-ELM and test ELM classifier sections, the ratio of subjects in the training set, validation set and test set is 90:90:20. The test set is generated randomly. It does not change during the KFS-ELM calculation. The training and validation sets were randomly divided for the construction of each ELM classifier. The training set is used to construct the ELM classifier. The validation set is used to evaluate and find the best ELM classifiers. In the pruning process, both the training and validation sets are used to evaluate the change in the accuracy of the ELM classifiers. Test set for evaluating changes in ELM classifier before and after pruning.

In the validation of key features section, the ratio of subjects in the training set, validation set and test set is 160:20:20.

### Construction of ELM Classifier

The training and validation sets are re-divided before each training of the classifier. The number of subjects and the proportion of categories in each set are kept constant. The number of input layer nodes is 4005, corresponding to all functional connections of the cerebrum. In our previous study, we found that the number of hidden layer nodes is positively correlated with the accuracy of the classifier in scenarios with small sample size and high feature dimensionality. The number of hidden nodes is set to 64,000 as the hardware performance allows. Due to the fact that this experiment involves two categories of data, the number of output nodes is set to 2. The convergence precision threshold *p* = 0.0015. The only things that need to be set manually are the number of hidden layer nodes and the convergence precision threshold. After the ELM classifier has been constructed, it will be tested using the test set.

### Pruning of the Input Layer

The pruning of the input layer is actually solving Equation 10 for *W^i^*, While ensuring that it satisfies the constraints of the set of inequality (12). As there are two variables *W^i^* and β*^j^* in Equation (10), the value of β*^j^* is fixed first when solving for *W^i^*. Due to the fact that the activation functions of the hidden layer nodes are nonlinear and the input layer is fully connected to the hidden layer, pruning the input layer nodes will result in a nonlinear variation in the output of the ELM classifier. Different pruning order or different number of nodes per pruning may screen different input layer nodes. Thus there are various ways of solving *W^i^*. In this experiment, *W^i^* is solved by zeroing the elements on the diagonal of *W^i^* one by one according to their order. that is, a new *W^i^* is generated when the accuracy of the ELM classifier is unchanged or improved (using the training and validation sets) after zeroing an element in *W^i^*. Also because of the nonlinearity of ELM, if one or more nodes are pruned in the input layer pruning process, the pruning process needs to be executed again until no input node can be pruned. For the same reason, if β*^j^* changes, it is also necessary to prune the input layer again.

### Pruning of the Hidden Layer

In the process of pruning the hidden layer nodes, the hidden layer nodes that have no effect on the accuracy of ELM classifier or improve the accuracy are pruned in turn. And then find β*^j^*. Because the ELM output is a linear summation of the hidden node outputs, no iterative computation is required. If any hidden layer node is pruned, the new β*^j^* and *W^i^* should be substituted into the process of “ELM classifier input layer pruning” to update *W^i^* again. If no hidden layer nodes are pruned, the pruning process of a single ELM classifier is completed.

### Obtain Key Features

A single *W^i^* corresponds to only part of the key features. ELM classifiers constructed by them are also less accurate. As more key features corresponding to W* are available, the higher the accuracy of the ELM classifier constructed using it will be. When the accuracy of the ELM classifier is no longer improved, the corresponding key features become complete.

### Validate Key Features

First, set the weights of non-key features equal to 0. A series of feature groups is formed by gradually excluding the features with the lowest weights from all features. We write the feature group as *F*_*i*_,*i* = [0,⋯,*s*], *s* is the highest weights of key features. *F_i_* is the feature group, containing all features whose weight *i*. *s+1* feature groups will be formed.

Then, redivide the training set, validation set and test set randomly. The ratio of training set, validation set and test set is 160:20:20, and the ratio of AD to CN in each set is 1:1. The data input to the ELM classifier is the features which are selected from each data set base on the feature group *F_i_*.

Finally, the ELM classifier constructed from each group of features is used to validate the feature group classification (diagnostic) ability. 10 times 10 fold cross-validation is performed for each feature group.

## Results

According to the KFS-ELM method, the accuracy of ELM classification constructed using the key feature set corresponding to W* is no longer improved when screening to the 17th group *W^i^*. The accuracy of the 17 ELM classifiers is shown in Table 1. The average training accuracy is 100%, the average verification accuracy is 97.84%, and the average test accuracy is 92.35%.

**TABLE 1:** Accuracy of ELM trained with full amount of features,

Classifier no.	Training accuracy (%)	Verification accuracy (%)	Test accuracy (%)
1	100.00	97.78	95.00
2	100.00	97.78	85.00
3	100.00	98.89	90.00
4	100.00	97.78	95.00
5	100.00	97.78	95.00
6	100.00	97.78	95.00
7	100.00	97.78	100.00
8	100.00	97.78	75.00
9	100.00	97.78	75.00
10	100.00	97.78	95.00
11	100.00	97.78	100.00
12	100.00	97.78	100.00
13	100.00	97.78	95.00
14	100.00	97.78	95.00
15	100.00	97.78	90.00
16	100.00	97.78	100.00
17	100.00	97.78	90.00%
Mean	100.00	97.84	92.35%

The accuracy of the 17 ELM classifiers after pruning is shown in Table 2. After pruning, the average training accuracy of all ELM classifiers remained 100%. the average validation accuracy increased from 97.84% to 99.8%. And the average test accuracy decreased from 92.35 to 84.71%. The number of input nodes and the number of hidden nodes are drastically reduced. The average number of input nodes decreases from 4005 to 106.24, which is 2.65% of the original number of input nodes. The average number of remaining hidden nodes decreased from 64,000 to 1042.53, which is 1.63% of the original number of hidden nodes.

**TABLE 2:** Number of nodes and accuracy of ELM classifier after pruning.

Classifier no.	Training accuracy (%)	Validation accuracy (%)	Test accuracy (%)	Number of input nodes screened	Number of hidden layer nodes screened
1	100.00	100.00	100.00	71	707
2	100.00	100.00	85.00	87	963
3	100.00	100.00	80.00	160	1,094
4	100.00	100.00	85.00	82	680
5	100.00	100.00	90.00	106	1,245
6	100.00	100.00	95.00	150	1,563
7	100.00	100.00	85.00	89	716
8	100.00	100.00	65.00	154	1,577
9	100.00	100.00	85.00	86	890
10	100.00	100.00	85.00	117	738
11	100.00	100.00	95.00	175	1,939
12	100.00	100.00	85.00	108	1,402
13	100.00	100.00	95.00	63	557
14	100.00	98.89	70.00	85	641
15	100.00	98.89	80.00	87	825
16	100.00	98.89	85.00	96	934
17	100.00	100.00	75.00	90	1,252
Mean	100.00	99.80	84.71	106.24	1,042.53

**FIGURE 2:**
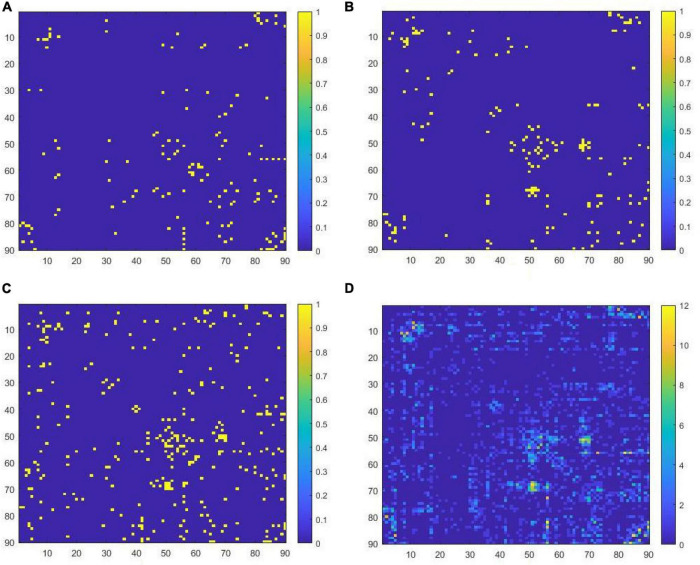
**(A)** Key features filtered in the first ELM classifier, **(B)** key features filtered in the second ELM classifier, **(C)** key features filtered in the third ELM classifier, **(D)** the concatenated set of all key features.

The key features corresponding to the pruned ELM classifier are shown in Figure 2 by the functional connectivity matrix. Figures 2A–C are the image representations of the key features (functional connectivity matrix) of the 1st, 2nd, and 3rd ELM classifier, respectively. Figure 2D is the image representation of W*. The horizontal and vertical coordinates of the image correspond to the serial numbers of the 90 brain regions of the AAL template. The colors of the points correspond to the weights of the key features. The total number of key features corresponding to W* is 920. where the highest weight is 12. That is, the intersection of 17 *W^i^* is empty.

Table 3 shows the performance of testing the classification (diagnosis) ability of key features with different weights versus full features for AD. Perform ten times 10 fold cross-validation and calculate the average of their accuracy rates. The ELM classifier trained using the full amount of features had a test accuracy of 95.33% with a standard deviation of 0.0035. The ELM classifier trained with all key features (920 features, 22.97% of the total number of features) had the highest test accuracy of 99.20% with a standard deviation of 0.0021. When the number of key features is 199 (4.97% of the total number of features), its test accuracy is 95.24%, which is only 0.09% lower than the accuracy of the full number of features. That is, the number of selected features was reduced from 4005 to 199, and the accuracy of the constructed ELM classifier test did not decrease significantly. When the number of selected features is 45 (1.12% of all features), its verification accuracy can still reach 100%, and the test accuracy is 87.84%. When the selected features are further reduced, the accuracy of the ELM classifier is also further reduced. When the number of selected features is 4 or 1, the ELM test accuracy is close to 50%. It is no longer practical to do classification using only these features.

**TABLE 3:** Accuracy of constructing ELM classifier with full amount of features or key features with different weights.

Criteria for feature selection	Amount of features	Selected features/Full amount of features (%)	Training accuracy (%)	Validation accuracy (%)	Test accuracy ± Standard deviation
Weight ≥ 0	4005	100.00	100.00	100.00	95.33% ± 0.0035
Weight ≥ 1	920	22.97	100.00	100.00	99.20% ± 0.0021
Weight ≥ 2	397	9.91	100.00	100.00	96.92% ± 0.0057
Weight ≥ 3	199	4.97	100.00	100.00	95.24% ± 0.0048
Weight ≥ 4	109	2.72	100.00	100.00	93.33% ± 0.0074
Weight ≥ 5	62	1.55	100.00	100.00	91.68% ± 0.0090
Weight ≥ 6	45	1.12	100.00	100.00	87.84% ± 0.0106
Weight ≥ 7	28	0.70	100.00	99.75	82.19% ± 0.0126
Weight ≥ 8	22	0.55	100.00	97.55	75.27% ± 0.0195
Weight ≥ 9	11	0.27	100.00	92.70	69.03% ± 0.0170
Weight ≥ 10	8	0.20	100.00	87.75	60.14% ± 0.0359
Weight ≥ 11	4	0.10	100.00	81.65	51.69% ± 0.0251
Weight ≥ 12	1	0.02	58.05	84.85	55.34% ± 0.0140

**FIGURE 3:**
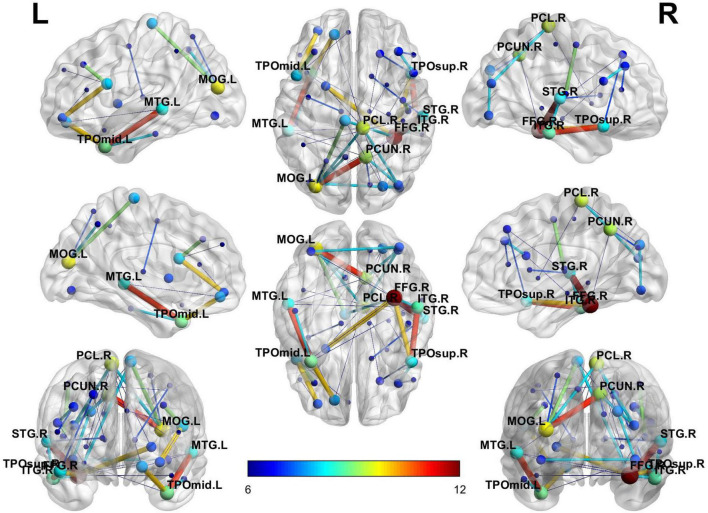
Full-view diagram of key features with weights greater than or equal to 6.

Considering the visualization effect, this paper takes 45 key features with weights greater than or equal to 6 as an example to show their distribution. Table 4 shows the features (functional connectivity) and their corresponding weights. Table 5 shows the brain regions corresponding to the features and their weights. Its weight is the sum of the weights of all functional connections involving that brain region. Figure 3 is a demonstration of these key features. The visualization tool used is BrainNet Viewer (Xia et al., 2013).

**TABLE 4:** The key features with weight value greater than or equal to 6.

Functional connectivity	Weight	Functional connectivity	Weight	Functional connectivity	Weight
FFG.R-STG.R (56–82)	12	MOG.R-PCUN.R (52–68)	8	ORBsup.L-MTG.L (5–85)	6
MOG.L-PCUN.R (51–68)	11	MOG.R-PCL.R (52–70)	8	ORBmid.L-IFGtriang.L (9–13)	6
TPOsup.R-ITG.R (84–90)	11	IOG.L-IOG.R (53–54)	8	IFGoperc.L-SFGmed.L (11–23)	6
MTG.L-TPOmid.L (85–87)	11	FFG.R-PCL.L (56–69)	8	IFGoperc.R-HIP.R (12–38)	6
ORBsup.L-TPOmid.L (5–87)	10	PCL.L-ITG.R (56–90)	8	SOG.R-PCL.L (50–69)	6
ORBmid.L-IFGoperc.L (9–11)	10	STG.R-ITG.R (82–90)	8	SOG.R-PCL.R (50–70)	6
FFG.R-PUT.L (56–73)	10	TPOmid.L-ITG.L (87–89)	8	IOG.L-FFG.R (53–56)	6
FFG.R-TPOsup.R (56–84)	10	PreCG.L-THA.L (1–77)	7	FFG.R-PCUN.R (56–68)	6
PreCG.R-HES.R (2–80)	9	SFGdor.R-TPOsup.R (4–84)	7	FFG.R-TPOsup.L (56–83)	6
MFG.L-IFGoperc.L (7–11)	9	MFG.R-IFGtriang.R (8–14)	7	PoCG.R-IPL.L 58–61)	6
MOG.L-PCL.L (51–69)	9	CUN.R-PCUN.R (46–68)	7	PCUN.R-MTG.L (68–85)	6
MFG.R-IFGoperc.R (8–12)	8	MOG.L-SPG.L (51–59)	7	PCL.R-PUT.L (70–73)	6
SOG.R-IOG.R (50–54)	8	CAU.R-STG.R (72–82)	7	PCL.R-PUT.R (70–74)	6
MOG.L-PCUN.L (51–67)	8	SFGdor.R-THA.R (4–78)	6	MTG.L-ITG.R (85–90)	6
MOG.L-PCL.R (51–70)	8	ORBsup.L-PCL.R (5–70)	6	MTG.R-TPOmid.L (86–87)	6

**TABLE 5:** The brain regions corresponding to the key features with weight greater than or equal to 6.

Brain region (serial No.)	Weight	Brain region (serial No.)	Weight	Brain region (serial No.)	Weight
FFG.R (56)	66	MOG.R (52)	16	CUN.R (46)	7
MOG.L (51)	43	IOG.R (54)	16	SPG.L (59)	7
PCL.R (70)	40	PUT.L (73)	16	CAU.R (72)	7
PCUN.R (68)	38	MFG.R (8)	15	THA.L (77)	7
TPOmid.L (87)	35	IFGoperc.R (12)	14	IFGtriang.L (13)	6
ITG.R (90)	33	IOG.L (53)	14	SFGmed.L (23)	6
MTG.L (85)	29	SFGdor.R (4)	13	HIP.R (38)	6
TPOsup.R (84)	28	PreCG.R (2)	9	PoCG.R (58)	6
STG.R (82)	27	MFG.L (7)	9	IPL.L (61)	6
IFGoperc.L (11)	25	HES.R (80)	9	PUT.R (74)	6
PCL.L (69)	23	PCUN.L (67)	8	THA.R (78)	6
ORBsup.L (5)	22	ITG.L (89)	8	TPOsup.L (83)	6
SOG.R (50)	20	PreCG.L (1)	7	MTG.R (86)	6
ORBmid.L (9)	16	IFGtriang.R (14)	7		

## Discussion

Comparing the ELM classifier before and after pruning (according to Tables 2, 3), a significant decrease in the number of input nodes was observed. The average number of input nodes decreased from 4005 to 106.24. Its corresponding number of key features accounts for 2.65% of the total number of features. According to the constraints of Inequality (12) and Inequality (13), removing any of the key nodes will lead to a decrease in the accuracy of the classifier. It shows that all these features are important. The KFS-ELM feature screening experiments yielded 17 groups of key features whose intersection is empty. This indicates that the key features that contribute differently in different classifiers. Therefore, the union of these key feature groups can describe AD more comprehensively.

The performance of the full amount of features and key features in building ELM classifiers was tested in the experiments. The ELM classifier trained using the full amount of features (4,005 features) has a test accuracy of 95.33%. The ELM classifier trained using all key features (920 features) has a test accuracy is 99.20%. It indicates that, training the ELM classifier with fewer features results in higher test accuracy, the 3,085 (4,005 − 920 = 3,085) features that were excluded had a negative effect in classification. Therefore, the KFS-ELM is effective in screening key features.

In this experiment, the weights assigned to the features by KFS-ELM are from 0 to 12, and the bigger the weights, the more important they are. In the testing section, we divided the features into 13 groups according to their weights and gradually excluded the group with the lowest feature weights to test the diagnostic ability of the remaining features for AD. For example, in Table 3, the row where “Weight ≥ 1” indicates the diagnostic ability of all key features (excluding features with weights equal to 0) for AD. Comparing the results of “Weight ≥ 0” and “Weight ≥ 1,” the features with weight equal to 0 play a negative role in AD diagnosis. The row where “Weight ≥ 2” indicates that the key features “Weight = 0” and “Weight = 1” are excluded. Comparing the results of “Weight ≥ 2” and “Weight ≥ 1,” we can judge the effect of the “Weight = 1” feature group on the diagnosis of AD. Adding the feature group of “Weight = 1” to the feature group of “Weight ≥ 2” increased the number of features by 523 (920 - 397), and the training and validation accuracy did not change, while the test accuracy increased by 2.28% (99.20 - 96.92%). That is, the 523 features(“Weight = 1”) contributed 2.28% to the test accuracy of AD. In this way, the effect of the features with weight = 10 on the diagnosis of AD can be shown by comparing the experimental results of “Weight ≥ 10” and “Weight ≥ 11.” The feature group with Weight = 10 contributed 6.10% (87.75 - 81.65%) to the validation accuracy and 8.45% (60.14 - 51.69%) to the test accuracy in AD diagnosis. According to this rule, the effect of feature groups with different weights on AD diagnosis was calculated based on the results in Table 3, as detailed in Table 6. In addition, according to Table 3, the accuracy of the training set was 58.05% when “Weight ≥ 12”, which means that the group of features with Weight = 12 is not enough to distinguish AD from normal people, so this result is not adopted as the basis for Table 6. Therefore, in Table 6, the impact of AD diagnosis can only be assessed for the feature groups with weights from 0 to 10.

**TABLE 6:** Effect of features with different weights on AD diagnosis.

Weight of feature group	Features amount	Contribution of feature group to validation accuracy (%)	Contribution of each feature to validation accuracy (%)	Contribution of feature group to test accuracy (%)	Contribution of each feature to test accuracy (%)
Weight = 10	4	6.10	1.5250	8.45	2.1125
Weight = 9	3	4.95	1.6500	8.89	2.9633
Weight = 8	11	4.85	0.4409	6.24	0.5673
Weight = 7	6	2.20	0.3667	6.92	1.1533
Weight = 6	17	0.25	0.0147	5.65	0.3324
Weight = 5	17	0.00	0.0000	3.84	0.2259
Weight = 4	47	0.00	0.0000	1.65	0.0351
Weight = 3	90	0.00	0.0000	1.91	0.0212
Weight = 2	198	0.00	0.0000	1.68	0.0085
Weight = 1	523	0.00	0.0000	2.28	0.0044
Weight = 0	3085	0.00	0.0000	-3.87	-0.0013

Each row in Table 6 indicates the effect of a key feature group on the accuracy of AD diagnosis for a given weight. The first column indicates the weights of the feature group. The second column indicates the number of features in the group. The third column indicates the contribution of the feature group to the validation accuracy. The fourth column indicates the average contribution of each feature in the group to the validation accuracy. The fifth column indicates the contribution of the feature group to the test accuracy. The sixth column indicates the average contribution of each feature in the group to the test accuracy.

According to Table 6, in terms of the contribution of single feature to the validation accuracy (column 4), basically the higher the weight of the feature, the more it contributes to the AD diagnosis (except weight = 9). In terms of the contribution of single feature to the test accuracy (column 6), basically the higher the weight of the feature, the more it contributes to the AD diagnosis (except weight = 8 and weight = 9). Basically, it can be considered that the higher weight the feature has, the greater contribution it makes to the AD diagnosis.

According to the pattern in column 2 of Table 6, the lower the weight of the feature group, the higher amount of features it contains. However, the group with a weight of 9 has fewer features than expected and the group with a weight of 8 has more features than expected. This also caused the contribution of features to diagnose AD deviated when the weight equals 8 and 9. We consider that this is due to the ELM classifier’s randomness in the utilization of features (refer to Figure 2). There are some deviations in the results of feature screening from the probability distribution of the features. Features with weights of 8, 9, 10, 11, and 12 account for 0.27, 0.07, 0.10, 0.07, and 0.02% of all features, respectively, and there are only 22 of these features in total, accounting for 0.55% of the total number of features. If there is a deviation of 0.1% in the feature screening, the results may deviate from the expected pattern, as it happens in Table 6 when the weights are equal to 8 and 9. When the number of features in the feature set is greater than or equal to 17 (0.42% of the total features), no such deviation occurs.

We have taken four references with similar work for comparison, as shown in Table 7. They all use functional connectivity as a feature measure, and they all screen for features as well. All the five studies used different feature screening methods. Bi et al. (2018) obtains feature sets by clustering, without assigning weights. It selects a class with the best classification performance from a large number of classes. Nguyen et al. (2019) set thresholds on the original values of the features. These values do not have a clear relationship with classification. Sadiq et al. (2021) and Hao et al. (2021) have in common that the features are given new weights by the feature screening method. They are both selected the top N features according to the weight. Hao et al. (2021) did not test the effects of different numbers of features on classification. It cannot be shown that there is a clear relationship between the weights of the features and the classification. Sadiq et al. (2021) used the ReliefF method to screen for features. This is a method that is widely used for feature screening. They tried 100, 200, 300, 400, 500 features for classification, respectively. The accuracy did not show a positive correlation with the number of features. Our experimental results show a clear positive correlation between the accuracy and the number of features.

**TABLE 7:** Comparison the performances with references.

References	DataSet AD:CN	Feature measures	Feature screening methods	Criteria for feature selection	Feature weights represent importance on classification	Classifier	Selected features	Selected features/All features(%)	Acc (%)
Bi et al., 2018	25:36	Funtional Connectivity (FC)	random neural network cluster	Selection of the best feature set from 11,000 times clustering	NO	Elam Neural Network	120	30.00	92.31
Nguyen et al., 2019	34:31	voxel-wise regional spontaneous, FC	t-test, SVM-RFE, LASSO	Thresholds for selecting features	NO	ELM	–	–	98.86
Sadiq et al., 2021	35:31	FC	ReliefF	Top n Features	NO	K-Nearest Neighbor	100	1.50	87.10
							200	3.00	93.50
							300	4.50	88.70
							400	6.00	91.90
							500	7.50	91.70
Hao et al., 2021	252:215 (none ADNI)	first-order neighborhood aggregation of FC	Weight-constrained lowrank Learning	Top n Features	NO	Multi-Kernel SVM	–	25.00	88.63
Proposed method	100:100	FC	KFS-ELM	Automatic generation	YES	ELM	45	1.12	87.84
							62	1.55	91.68
							109	2.72	93.33
							199	4.97	95.24
							397	9.91	96.92
							920	22.97	99.20

In summary, the KFS-ELM method can rationally identify the weights of key features. The higher the weights of the features, the greater the impact in AD diagnosis.

Extreme learning machine is linearly transformed between the hidden and output layers. The KFS-ELM method has achieved good performance based on ELM. Some other representative RFNN methods may achieve high classification accuracies in scenarios with high-dimensional features, such as RVFL, where it is non-linearly transformed between the hidden and output layers. In the future, we will attempt to use the idea of key feature screening for research on the RVFL. We will also apply the proposed method to explore patterns in AD-like diseases or other brain sciences.

## Conclusion

The experimental results and discussion analysis showed that, the KFS-ELM method can effectively screen key features related to the diagnosis of AD, and can assign rational weights to the features to identify their importance for the diagnosis of AD. The KFS-ELM can be used to construct better classifiers for the diagnosis of AD, and can also be used as a feature analysis tool to study the patterns inherent in the brains of AD patients. We consider that the KFS-ELM is also applicable to the classification and the study of feature patterns of the other objects with high feature dimensions, even with small sample sizes.

## Data Availability Statement

The original contributions presented in the study are included in the article/Supplementary Material, further inquiries can be directed to the corresponding author.

## Ethics Statement

The studies involving human participants were reviewed and approved by ADNI. All the fMRI data used in this manuscript are from LONI’s ADNI database ADNI2 project. It can be downloaded from http://adni.loni.usc.edu. All ADNI participants signed an informed consent form at the time of fMRI and related information collection, and the protocol for consent was approved by the institutional review board at each site. The ethical approval was obtained by ADNI and can be found at http://www.loni.usc.edu/ADNI/. All studies in this manuscript were conducted in accordance with the relevant guidelines.

## Author Contributions

JL and WZ contributed to the conception of the study. JL performed the experiment and data analyses and wrote the manuscript. JL and LZ contributed significantly to the analysis and manuscript preparation. YS helped to perform the analysis with constructive discussions. All authors contributed to the article and approved the submitted version.

## Conflict of Interest

The authors declare that the research was conducted in the absence of any commercial or financial relationships that could be construed as a potential conflict of interest.

## Publisher’s Note

All claims expressed in this article are solely those of the authors and do not necessarily represent those of their affiliated organizations, or those of the publisher, the editors and the reviewers. Any product that may be evaluated in this article, or claim that may be made by its manufacturer, is not guaranteed or endorsed by the publisher.

## Footnotes

1
http://rfmri.org/DPARSF

2
https://www.fil.ion.ucl.ac.uk/spm/software/spm12/

## References

[B1] Bi X. A. Jiang Q. Sun Q. Shu Q. Liu Y. (2018). Analysis of Alzheimer’s disease based on the random neural network cluster in fMRI. *Front. Neuroinform.* 12:60. 10.3389/fninf.2018.00060 30245623 PMC6137384

[B2] Buckner R. L. Sepulcre J. Talukdar T. Krienen F. M. Liu H. Hedden T. (2009). Cortical hubs revealed by intrinsic functional connectivity: mapping, assessment of stability, and relation to Alzheimer’s disease. *J. Neurosci.* 29 1860–1873. 10.1523/JNEUROSCI.5062-08.2009 19211893 PMC2750039

[B3] Chyzhyk D. Savio A. Graña M. (2015). Computer aided diagnosis of schizophrenia on resting state fMRI data by ensembles of ELM. *Neural Netw.* 68 23–33. 10.1016/j.neunet.2015.04.002 25965771

[B4] Guo P. Chen C. P. Sun Y. (1995). “An exact supervised learning for a three-layer supervised neural network,” in *Proceedings of the1995 International Conference on Neural Information Processing*, (Beijing: Publishing House of Electronics Industry), 1041–1044.

[B5] Hao X. K. An Q. J. Jiang T. Guo Y. C. Li J. Shi S. (2021). “Multi-template neuroimaging feature selection using weight-constrained low-rank learning for alzheimer’s disease classification,” in *Proceedings of the 2021 36th Youth Academic Annual Conference of Chinese Association of Automation (YAC)*, Nanchang, 173–178. 10.1109/YAC53711.2021.9486487

[B6] Huang G. Song S. J. Gupta J. N. D. Wu C. (2014). Semi-supervised and unsupervised extreme learning machines. *IEEE Trans. Cybern.* 44 2405–2417. 10.1109/tcyb.2014.2307349 25415946

[B7] Huang G. B. Babri H. A. (1998). Upper bounds on the number of hidden neurons in feedforward networks with arbitrary bounded nonlinear activation functions. *IEEE Trans. Neural Netw.* 9 224–229. 10.1109/72.655045 18252445

[B8] Huang G. B. Chen L. (2007). Convex incremental extreme learning machine. *Neurocomputing* 70 3056–3062. 10.1016/j.neucom.2007.02.009

[B9] Huang G. B. Chen L. (2008). Enhanced random search based incremental extreme learning machine. *Neurocomputing* 71 3460–3468. 10.1016/j.neucom.2007.10.008

[B10] Huang G. B. Wang D. H. Lan Y. (2011). Extreme learning machines: a survey. *Int. J. Mach. Learn. Cybern.* 2 107–122. 10.1007/s13042-011-0019-y

[B11] Huang G. B. Zhou H. M. Ding X. J. Zhang R. (2012). Extreme learning machine for regression and multiclass classification. *IEEE Trans. Syst. Man. Cybern.* 42 513–529. 10.1109/tsmcb.2011.2168604 21984515

[B12] Huang G. B. Zhu Q. Y. Siew C. K. (2004). “Extreme learning machine: a new learning scheme of feedforward neural networks,” in *Proceedings of the IEEE International Joint Conference on Neural Networks* (Budapest: IEEE), 985–990. 10.1109/IJCNN.2004.1380068

[B13] Huang G. B. Zhu Q. Y. Siew C. K. (2006). Extreme learning machine: theory and applications. *Neurocomputing* 70 489–501. 10.1016/j.neucom.2005.12.126

[B14] Huang Z. Yu Y. Gu J. Liu H. (2016). An efficient method for traffic sign recognition based on extreme learning machine. *IEEE Trans. Cybern.* 47 920–933. 10.1109/TCYB.2016.2533424 26992185

[B15] Kazemifar S. Drozd J. J. Rajakumar N. Borrie M. J. Bartha R. (2014). Automated algorithm to measure changes in medial temporal lobe volume in Alzheimer disease. *J. Neurosci. Meth.* 227 35–46. 10.1016/j.jneumeth.2014.01.033 24518149

[B16] Kim J. Kim J. Jang G. J. Lee M. (2017). Fast learning method for convolutional neural networks using extreme learning machine and its application to lane detection. *Neural Netw.* 87 109–121. 10.1016/j.neunet.2016.12.002 28110106

[B17] Lama R. K. Kwon G. R. (2021). Diagnosis of Alzheimer’s disease using brain network. *Front. Neurosci.* 15:605115. 10.3389/fnins.2021.605115 33613178 PMC7894198

[B18] Liu X. Zeng Y. Zhang T. L. Xu B. (2016). Parallel brain simulator: a multi-scale and parallel brain-inspired neural network modeling and simulation platform. *Cogn. Comput.* 8 967–981. 10.1007/s12559-016-9411-y

[B19] Liu X. Y. Gao C. H. Li P. (2012). A comparative analysis of support vector machines and extreme learning machines. *Neural Netw.* 33 58–66. 10.1016/j.neunet.2012.04.002 22572469

[B20] Malik A. K. Ganaie M. A. Tanveer M. Suganthan P. N. Alzheimer’s Disease, and Neuroimaging Initiative (2022). Alzheimer’s disease diagnosis via intuitionistic fuzzy random vector functional link network. *IEEE Trans. Comput. Soc. Syst.* (in press). 1–12. 10.1109/TCSS.2022.3146974

[B21] Miche Y. Sorjamaa A. Bas P. Simula O. Jutten C. Lendasse A. (2010). OP-ELM: optimally pruned extreme learning machine. *IEEE Trans. Neural Netw.* 21 158–162. 10.1109/TNN.2009.2036259 20007026

[B22] Miche Y. van Heeswijk M. Bas P. Simula O. Lendasse A. (2011). TROP-ELM: a double-regularized ELM using LARS and Tikhonov regularization. *Neurocomputing* 74 2413–2421. 10.1016/j.neucom.2010.12.042

[B23] Nguyen D. T. Ryu S. Qureshi M. N. I. Choi M. Lee K. H. Lee B. (2019). Hybrid multivariate pattern analysis combined with extreme learning machine for Alzheimer’s dementia diagnosis using multi-measure rs-fMRI spatial patterns. *PLoS One* 14:e0212582. 10.1371/journal.pone.0212582 30794629 PMC6386400

[B24] Pao Y. H. Park G. H. Sobajic D. J. (1994). Learning and generalization characteristics of the random vector functional-link net. *Neurocomputing* 6 163–180. 10.1016/0925-2312(94)90053-1

[B25] Richhariya B. Tanveer M. Rashid A. H. Alzheimer’s Disease Neuroimaging Initiative (2020). Diagnosis of Alzheimer’s disease using universum support vector machine based recursive feature elimination (USVM-RFE). *Biomed. Signal. Proces.* 59:101903. 10.1016/j.bspc.2020.101903

[B26] Rong H. J. Ong Y. S. Tan A. H. Zhu Z. X. (2008). A fast pruned-extreme learning machine for classification problem. *Neurocomputing* 72 359–366. 10.1016/j.neucom.2008.01.005

[B27] Rubinov M. Sporns O. (2010). Complex network measures of brain connectivity: uses and interpretations. *Neuroimage* 52 1059–1069. 10.1016/j.neuroimage.2009.10.003 19819337

[B28] Sadiq A. Yahya N. Tang T. B. (2021). “Diagnosis of Alzheimer’s disease using pearson’s correlation and relieff feature selection approach,” in *Proceedings of the 2021 International Conference on Decision Aid Sciences and Application (DASA)*, Bahrain, 578–582. 10.1109/DASA53625.2021.9682409

[B29] Schmidt W. F. Kraaijveld M. A. Duin R. P. (1992). “Feed forward neural networks with random weights,” in *Proceedings of the International Conference on Pattern Recognition*, Netherlands, 1–1.

[B30] Sharma R. Goel T. Tanveer M. Dwivedi S. Murugan R. (2021). FAF-DRVFL: fuzzy activation function based deep random vector functional links network for early diagnosis of Alzheimer disease. *Appl. Soft Comput.* 106:107371. 10.1016/j.asoc.2021.107371

[B31] Suganthan P. N. Katuwal R. (2021). On the origins of randomization-based feedforward neural networks. *Appl. Soft Comput.* 105:107239. 10.1016/j.asoc.2021.107239

[B32] Supekar K. Menon V. Rubin D. Musen M. Greicius M. D. Sporns O. (2008). Network analysis of intrinsic functional brain connectivity in Alzheimer’s Disease. *PLoS Comput. Biol.* 4:e1000100. 10.1371/journal.pcbi.1000100 18584043 PMC2435273

[B33] Susanne G. M. Michael W. W. Leon J. T. Ronald C. P. Clifford R. J. William J. (2005). Ways toward an early diagnosis in Alzheimer’s disease: the Alzheimer’s Disease Neuroimaging Initiative (ADNI). *Alzheimers Dement.* 1 55–66. 10.1016/j.jalz.2005.06.003 17476317 PMC1864941

[B34] Tang J. X. Deng C. W. Huang G. B. Zhao B. J. (2015). Compressed-domain ship detection on spaceborne optical image using deep neural network and extreme learning machine. *IEEE Trans. Geosci. Remote* 53 1174–1185. 10.1109/tgrs.2014.2335751

[B35] Tanveer M. Richhariya B. Khan R. U. Rashid A. H. Khanna P. Prasad M. (2020). Machine learning techniques for the diagnosis of Alzheimer’s disease: a review. *ACM Trans. Multim. Comput.* 16 1–35. 10.1145/3344998

[B36] Tzourio-Mazoyer N. Landeau B. Papathanassiou D. Crivello F. Etard O. Delcroix N. (2002). Automated anatomical labeling of activations in SPM using a macroscopic anatomical parcellation of the MNI MRI single-subject brain. *Neuroimage* 15 273–289. 10.1006/nimg.2001.0978 11771995

[B37] Wang S. G. Deng C. W. Lin W. S. Huang G. B. Zhao B. J. (2016). NMF-based image quality assessment using extreme learning machine. *IEEE Trans. Cybern.* 47 1–12. 10.1109/TCYB.2015.2512852 26863686

[B38] Xia M. R. Wang J. H. He Y. (2013). BrainNet viewer: a network visualization tool for human brain connectomics. *PLoS. One* 8:e68910. 10.1371/journal.pone.0068910 23861951 PMC3701683

[B39] Yan C. G. Zang Y. F. (2010). DPARSF: a MATLAB toolbox for “pipeline” data analysis of resting-state fMRI. *Front. Syst. Neurosci.* 4:13. 10.3389/fnsys.2010.00013 20577591 PMC2889691

[B40] Zhou J. Gennatas E. D. Kramer J. H. Miller B. L. Seeley W. W. (2012). Predicting regional neurodegeneration from the healthy brain functional connectome. *Neuron* 73 1216–1227. 10.1016/j.neuron.2012.03.004 22445348 PMC3361461

